# Mortality advantage among migrants according to duration of stay in France, 2004–2014

**DOI:** 10.1186/s12889-019-6652-1

**Published:** 2019-03-21

**Authors:** Matthew Wallace, Myriam Khlat, Michel Guillot

**Affiliations:** 1 Demography Unit, Department of Sociology, Stockholm University, Stockholm, Sweden; 2 Institut national d’études démographiques, French National Demographic Institute, 133 Boulevard Davout, 75020 Paris, France; 3 Population Studies Center, University of Pennsylvania, 242 McNeil Building, Philadelphia, PA19104 USA

**Keywords:** Migrant mortality advantage, Healthy migrant effect, All-cause mortality, Selection, Acculturation, Assimilation, Gender, Duration of stay, Length of residence

## Abstract

**Background:**

The migrant mortality advantage is generally interpreted as reflecting the selection of atypically healthy individuals from the country of origin followed by the wearing off of selection effects over time, a process theorised to be accelerated by progressive and negative acculturation in the host country. However, studies examining how migrant mortality evolves over duration of stay, which could provide insight into these two processes, are relatively scarce. Additionally, they have paid little attention to gender-specific patterns and the confounding effect of age. In this study, we analyze all-cause mortality according to duration of stay among male and female migrants in France, with a particular focus on the role of age in explaining duration of stay effects.

**Methods:**

We use the *Échantillon Démographique Permanent* (Permanent Demographic Sample; EDP), France’s largest socio-demographic panel and a representative 1% sample of its population. Mortality was followed-up from 2004 to 2014, and parametric survival models were fitted for males and females to study variation in all-cause mortality among migrants over duration of stay. Estimates were adjusted for age, duration of stay, year, education level and marital status. Duration of stay patterns were examined for both open-ended and fixed age groups.

**Results:**

We observe a *migrant mortality advantage,* which is most pronounced among recent arrivals and converges towards the mortality level of natives with duration of stay. We show this pattern to be robust to the confounding effect of age and find the pattern to be consistent among males and females.

**Conclusions:**

Our novel findings show an intrinsic pattern of convergence of migrant mortality towards native-born mortality over time spent in France, independent from the ages at which mortality is measured. The consistent pattern in both genders suggests that males and females experience the same processes associated with generating the migrant mortality advantage. These patterns adhere to the selection-acculturation hypothesis and raise serious concerns about the erosion of migrant health capital with increasing exposure to conditions in France.

**Electronic supplementary material:**

The online version of this article (10.1186/s12889-019-6652-1) contains supplementary material, which is available to authorized users.

## Background

One of the most enduring findings in the social sciences literature is that of the *migrant mortality advantage*, a phenomenon defined *as* lower mortality among international migrants relative to native populations in high-income host countries [1–15]. The generally accepted – *if rarely empirically observed* – explanation for this pattern is the positive selection of atypically healthy and robust individuals from their origin countries [12]. Selection effects are theorized to be at their strongest just after migrants have arrived and wear off with time spent in the host country. This selection process may be accelerated by exposure to adverse social conditions and/or a progressive acculturation to prevailing beliefs, attitudes, and behaviors of the host society, which causes a shift in the disease patterns of migrants towards that of the host population [16]. However, it should be stated that this wearing off of selection effects can occur in the absence of any acculturation processes [17]. In lieu of comparable and reliable mortality data between migrants and natives in their origin countries (to directly examine selection), and rich longitudinal information on migrant health behaviors (to investigate acculturation), analyzing all-cause mortality according to duration of stay in the host country can offer insight into the *migrant mortality advantage* and its main explanations [18].

Studies into exactly how the *migrant mortality advantage* varies over duration of stay are scarce. In four of the studies we identified, there was no apparent effect of duration of stay on mortality [5, 18–21]. In the remaining six studies, there was some convergence in mortality towards the levels of natives over duration of stay [11, 14, 16, 22–24]. However, across these six studies, there were substantial differences in how quickly migrant mortality converged (and whether mortality converged fully), whether it was converging from an initial point of advantage or disadvantage, and according to the region or country of birth. Additionally, duration of stay was inconsistently defined across the studies, thus drawing comparisons across studies was difficult. Lastly, although several studies have investigated mortality from specific causes of death [25–31], their focus has been on determining the roles of genetic predisposition and environment in disease etiology [16, 32], rather than the main explanations of an overall mortality advantage. Therefore, our understanding of the patterns behind this low mortality remains unclear.

In this study, we use the largest individual-level longitudinal data source in France, which is representative in terms of both population structure and mortality patterns by age and sex to national estimates from the French National Institute of Statistics and Economic Studies (*Institut National de la Statistique et des Études Économiques, INSEE*). Our overarching goal is to determine whether the *migrant mortality advantage* is pronounced among migrants who have recently arrived and converges with duration of stay, consistent with the proposed primary explanations of this phenomenon. To frame our research we present two specific research questions:

1. *Does the relationship between duration of stay and mortality vary by sex?*

Only two of the cited studies examined sex differences by duration of stay [11, 23] despite the fact that being male or female is theorized to play some role in whether migrants experience a mortality advantage or not [2, 9, 33]. In a recent review of the French literature, a striking feature was the relative mortality advantage experienced by males as compared with the disadvantage experienced by females [33]. The authors posited that this could relate to a weaker selection effect among female migrants who move for family reunification [2, 9, 33] and are therefore only admitted as “dependent” wives and not as “independent” women who have chosen to migrate [34]. Such an assertion is relevant, but needs to be considered carefully with respect to specific arrival cohorts,  gender norms in the origin and host countries and sex-specific integration processes. Explicitly examining sex differentials in the migrant mortality advantage according to duration of stay represents the first key contribution of our study.

(2) *Is there an intrinsic duration of stay pattern which is independent of age?*

Additionally, in most of the studies that we cited, the analyses adjusted for age and duration of stay but the ages over which mortality was measured were not fixed [11, 14, 19, 21, 23, 24]; it was only examined over wide or open-ended intervals. Only three of the studies fixed age into narrow bands of 15-years or less [16, 18, 22]. It is crucial when investigating the effect of duration to fix age into narrow bands, rather than simply adjusting for it, otherwise it becomes to difficult to disentangle whether the observed patterns are caused by age or duration. The reasons for this center around two demographic expectations. First, the average age of migrants who have lived in the host country for a short time will be lower than that of migrants who have lived in the host country a long time. Second, as age *increases* mortality levels *increase* but variability in relative terms around these levels *decreases*. Consequently, mortality differentials at older ages tend to be smaller than at younger ages. In the absence of this knowledge, if one was to observe a larger mortality advantage in a shorter duration group than in a longer one among adult migrants in a study using wide or open-ended age bands, one might interpret this a duration effect when it could equally be an age effect [35, 36]. Thus, fixing the ages at which mortality is measured and letting duration of stay vary addresses this problem, allowing us interpret the patterns as an intrinsic effect of duration of stay. This represents the second main contribution of our study.

## Methods

The French Permanent Demographic Sample (*Échantillon Démographique Permanent*; EDP) is France’s largest socio-demographic panel and a representative 1% sample of the French population. It combines vital event information from official civil registers (birth, deaths, and marriages) with census data. Eligibility for the sample is based on date of birth (being born on one of four dates in one of January, April, July, or October). The EDP is a dynamic sample that is refreshed over time. New people can enter the sample through being born in, or moving to, France (and being born on a sample date) and leave the sample through death or leaving the country). EDP members’ records are updated with new information and are kept after their death.

Although the EDP has been active since 1968, we only follow individuals from 2004 as the year of arrival question was only asked consistently at censuses from this point. However, for 1 month (October – the original sampling month of the EDP prior to the expansion of the sample in 2004) we also benefit from direct and indirect retrospective information on year of arrival before 2004 (at least at censuses in which the question was included). Unfortunately, retrospective information was not linked for the other sampling months after the 2004 expansion of the sample.

To construct our main variable of interest – duration of stay – we relied upon two questions: year of arrival (“*If you are foreign-born, what year did you arrive in France?*”) and previous place of residence (“*Where did you live on date x?*”). The rate of non-response in these two questions was substantial (20%) and selective. Fitting a logistic regression model with non-response as our explanatory variable and after adjustment for age, sex and education, we found that those who did not respond were more likely to die (**OR: 1.30**; 95% CIs: 1.20–1.41). Consequently, we decided to limit our sample to those born in 1 month – October – due to availability of prior censuses and civil register information. For these migrants we could rely on retrospective data from three of five exhaustive censuses on year of arrival (1968, 1975, and 1999), past place of residence, and census presence to eliminate this undesirable level of non-response. This also provided the opportunity to validate information provided by migrants in the year of arrival question, as we could corroborate the date provided for those with long durations with these additional indicators of arrival. In short, we observed a reassuring level of consistency between dates. Given the EDP’s sampling method, the omission of the other sampling months should not have a substantive impact on results (except for the loss of some statistical power in our regression models).

Duration of stay was generated by subtracting year of arrival from year of first enumeration at a census point for each person. We gave natives an arbitrary value “88,888”. We categorized the variable into bands 0–5, 5–10, 10–15, 15–20, 20–30, 30–40, 40–50, 50–60 and 60 years and over.

We estimated adult mortality for ages 20+ by sex fitting continuous-time survival models in which ***u***_***i***_(***t***) denotes the hazard (or ‘force’) of mortality for individual ***i*** at age ***t*** and ***u***_**0**_(***t***) denotes the baseline hazard (risk of mortality by age which follows a Gompertz distribution i.e. an exponential increase in mortality with age). ***x***_***ij***_(***t***) represents our vector of explanatory covariates.

$$ {u}_i(t)={u}_0(t)\times \mathit{\exp}\left\{\sum \limits_j{\beta}_j{x}_{ij}(t)\right\} $$

In the baseline model, the vector of covariates was age (the baseline hazard), duration of stay, and year of onset of risk. The latter was a categorical variable from 2004 to 2013. In the final model, the vector of covariates was expanded to include education level and marital status. Education level at censuses was categorized according to the International Standard Classification of Education (ISCED) and coded into categories: *less than primary*, *primary*, *secondary* (secondary 1st and 2nd cycle) and *tertiary* (post-secondary to pre-university and above). Marital status was taken as provided from the census: *single*, *married*, *divorced* and *widowed*.

**Fig. 1:**
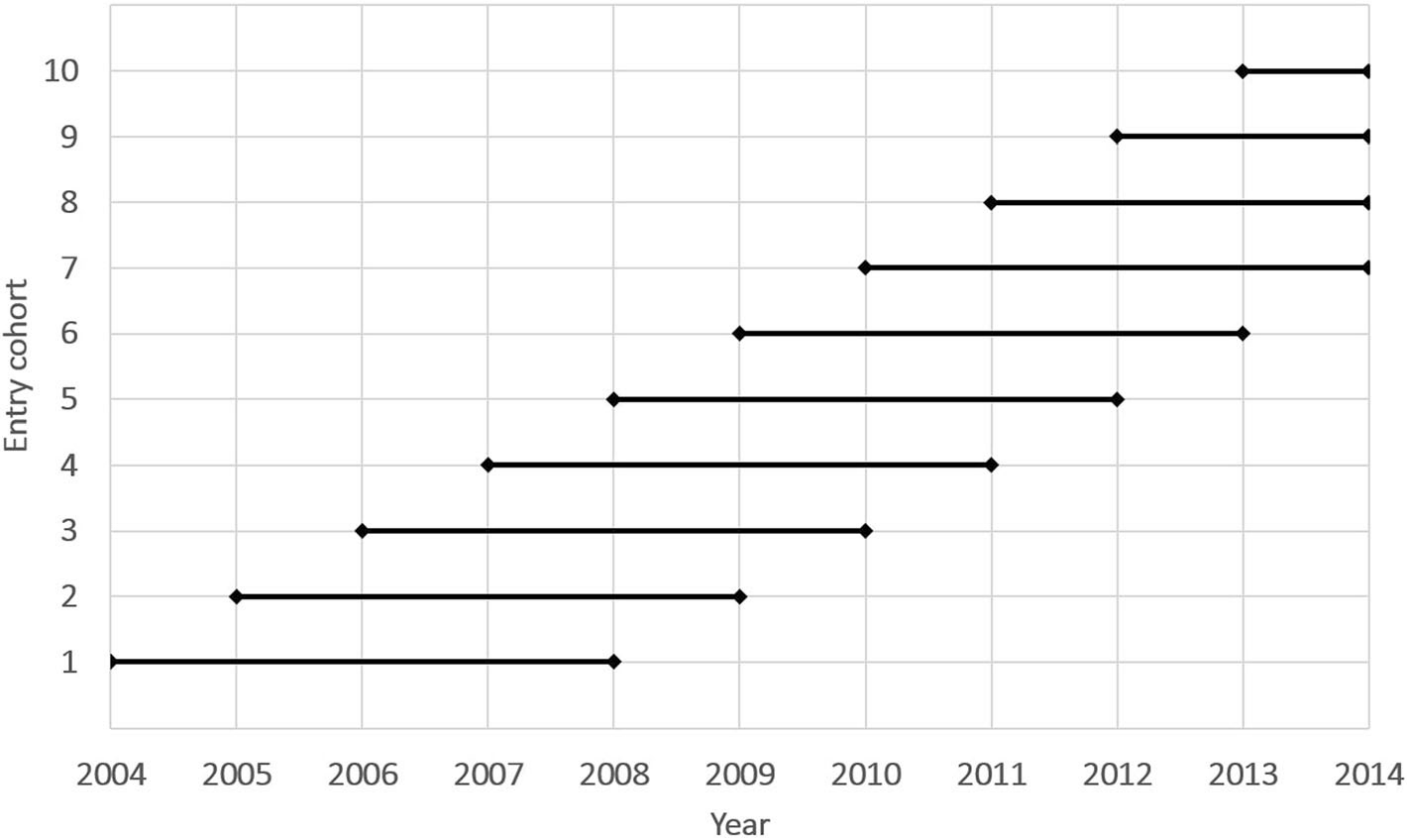
Study design: onset of risk and follow-up periods of annual entry cohorts

Figure 1 presents the study design. Our study period began in 2004 (the onset of the first rolling census) and ended in October 2014, the latest point for which we had death data and final collection year of the second rolling census. Individuals were considered to be “at risk” from the year they were enumerated at a rolling census point for the first time between 2004 and 2013 as long as they were aged 20+. We followed 10 annual entry cohorts (the first one in 2004 [entry cohort 2004; Fig. 1] and final one in 2013 [entry cohort 2013; Fig. 1]). We followed these cohorts for 5-years [cohorts 2004–9], or up until the end of the study period [cohorts 2010–13], whichever came first. We intentionally restricted the length of follow-up in earlier cohorts to limit the impact of ‘censoring bias’ (underestimation of migrant mortality due to an inability to remove from the risk set any migrants who have left the host country [5, 10, 37, 38]). Limiting follow-up ensured that any bias introduced through an inability to censor leavers was minimized. We note that we experimented with several different  follow-up lengths, but this did not have a substantive impact upon our main findings; 5-years represents a comprimise between sufficient power and minimizing censoring bias. Individuals in each entry cohort were followed until their death or until the end of study.

Our analytical strategy entails first examining the relationship between duration of stay and mortality over an open-ended age interval (20+) as is common in the literature. This analysis serves to show the difficulties we encounter in trying to isolate the role of duration of stay in migrant mortality patterns when age is not fixed. Next, we fix age into narrow 10-year bands (60–70 and 70–80) but continue to allow duration of stay to vary to see if we can identify an intrinsic duration effect (*research Q2*). The analyses are conducted separately for male and females (*research Q1*) in Stata 15.1. We compare all-cause mortality of 29,118 foreign-born males (1,188 deaths) with 175,842 native males (10,368 deaths) and 30,959 foreign-born females (1,063 deaths) with 193,288 native females (10,395 deaths).

Additional file 1: Tables S5–S7 also include additional descriptive information on the composition of each duration group according to characteristics: region of birth, education level, age, year of arrival, and age at arrival. These descriptives will help us to interpret any patterns we might observe. Age at arrival is particularly important, as the mortality of migrants is the summation of both their duration of stay and age at arrival. Given age is such a strong determinant of mortality, this age/duration of stay/age at arrival issue constitutes an extension of the non-identifiability problem of age/period/cohort models in the study of time trends [25]. Age at arrival is associated with variation in selection. Case in point, a migrant arriving aged 10 with his or her parents may not be subjected to the same level of selection as a migrant arriving aged 25 who has chosen to move to the host country for work. A recent study documented excess mortality among child migrants (aged less than 20-years) in the U.S., France and the U.K. The authors argued that these migrants will play a large role in observed relationships between duration of stay and mortality and that for them, a lack of positive selection may play a more crucial role than duration of stay effects [39]. Similarly, a study in Sweden combined information on age at arrival and duration of stay to demonstrate an excess mortality among migrants arriving before age 18 and only a moderate duration effect in specific migrant groups [21]. We thus pay close attention to age at arrival when interpreting findings.

## Results

**Fig. 2:**
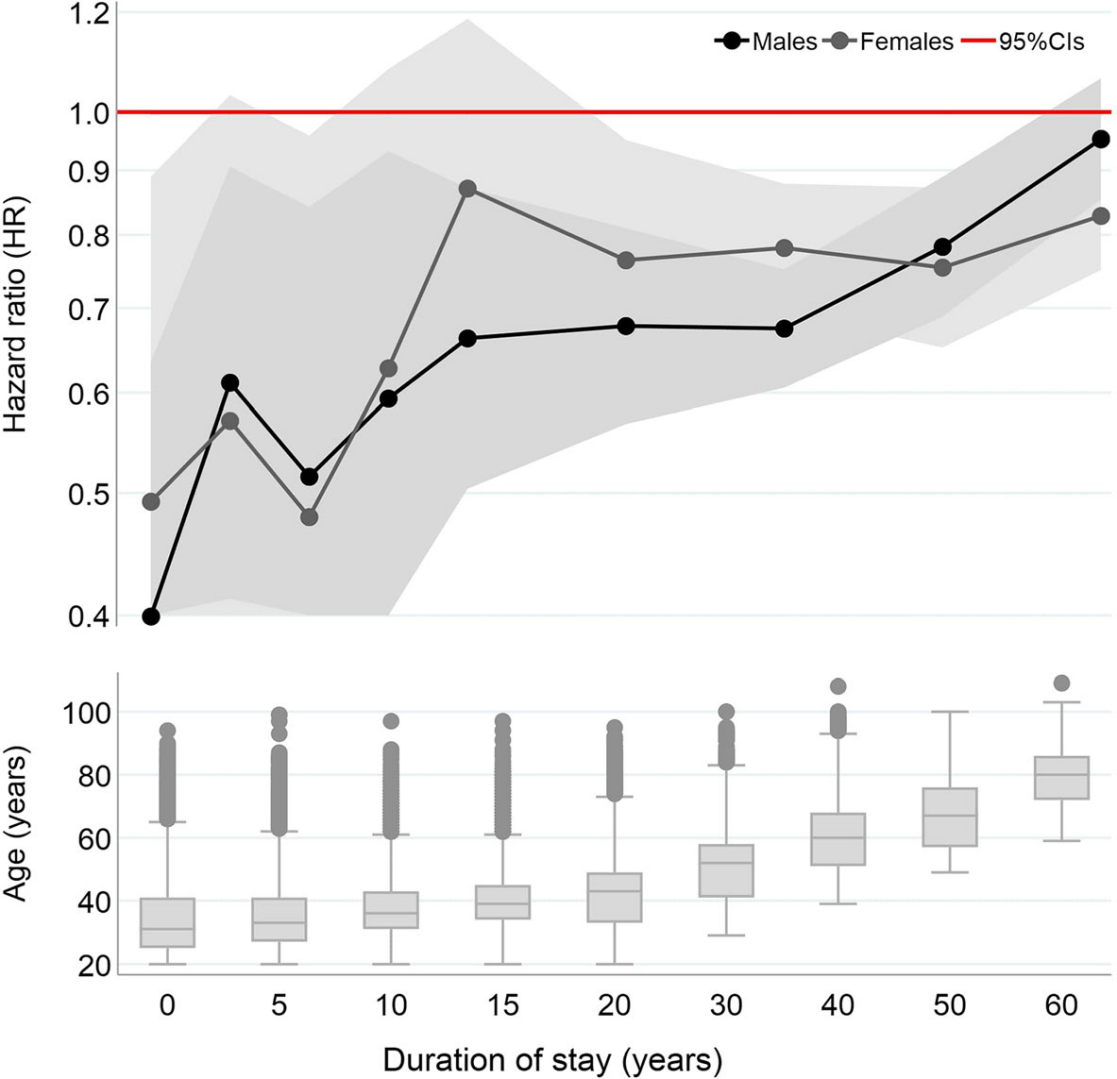
Hazard ratios (log) for all-cause mortality among male and female migrants over duration of stay, combined with box plots of age for each duration category, ages 20+. *Survival analyses adjusted for age, baseline year of entry, education level, and marital status. See Tables S1 and S2 of Additional file* *1* *for regression tables*

Figure 2 presents hazard ratios (HRs) for all-cause mortality by duration of stay among adult migrants (aged 20+) relative to natives born in France. The hazard ratios are displayed from the final model additionally adjusting for educational level and marital status (regression tables for the baseline and final model are both available in the Additional file 1: Tables S1 and S2). For each duration category, we include boxplots detailing the age composition of migrants to highlight the close relationship between the two variables. For both sexes, we observe a convergence with duration, though the pattern is more firmly established among males. HRs are pronounced among migrants who have arrived in the past 5-years (males **HR = 0.39**; 95% CIs 0.25–0.63; females **HR = 0.49**; 95% CIs 0.27–0.89) and move closer to 1 with duration of stay. For males, mortality has converged by 60 years + (**HR = 0.95**; 95% CIs 0.85–1.06), but among females a *migrant mortality advantage* persists (**HR = 0.83**; 95% CIs 0.75–0.91). Our post-estimation tests show that mortality in the shortest duration of stay band is statistically significantly different from the longest duration of stay band for males (**HR = 2.39**; *p* < 0.05) and for females (**HR = 1.68**; *p* < 0.05).

Taken at face value, the pattern is consistent with what would be expected in the presence of selection effects and progressive acculturation with increasing duration of stay. This is further corroborated by the additional compositional information in Additional file 1: Tables S5 (males) and S6 (females). We find that in the duration categories where the advantage is most pronounced (< 15-years), migrants are more highly educated (this is, of course, adjusted for in the models) and almost half arrive from Sub-Saharan Africa and Other Europe (migrant streams known to be highly skilled and educated) [40]. These migrants also arrive at prime working ages (as is evidenced by information on the age at arrival) with few arriving younger than age 18 (the age at which it is no longer possible for non-EU migrant children to arrive on family reunification visas). Moreover, the older duration categories are typically associated with migration from Algeria (a colony until 1962), Morocco and Tunisia (which were protectorates until 1955) and Southern Europe (older migration streams in France that were known to be less skilled and lower educated) [40].

However, Additional file 1: Tables S5 and S6 provide us with two important pieces of information that lead us to question this interpretation. First, average age at arrival decreases with duration of stay. This indicates that many migrants in longer duration bands arrived in France as children. Second, average age increases with duration of stay (we also refer readers to the box plots in Fig. 2). It therefore becomes impossible to determine whether the observed patterns are a generated by duration of stay (a wearing off of selection effects over time accelerated by acculturation), age at arrival (weaker selection in longer durations as more migrants arrived as children), or age (a reduction in the variability of mortality levels in longer durations bands as migrants are older).

Therefore, to assess the role of duration of stay in the migrant mortality advantage, we extended the analysis by fitting the same models but fixing age at two age groups at baseline: 60–70 and 70–80 years. This has three consequences. First, age no longer increases over the duration of stay categories. To elaborate, duration of stay can continue to vary from 0 to 70 [in the first age band] and 0 to 80 [in the second age band], but the age of migrants can only vary within a very narrow interval (from 60 to 70 [in the first age band] and 70 to 80 [in the second age band]). Second, by fixing age, we create a near perfect correlation between age at arrival and duration of stay. Then, if we assume a constant selection by age at arrival past age 18 (i.e. the age at which arriving on a family reunification visa as a child is no longer possible), then we propose that the effects we observe can be considered as isolated duration of stay effects. Of course, the two longest duration of stay bands are comprised of migrants who arrived exclusively as children, so the same is not true for migrants in these two categories. Third, because variability in mortality is lower in the two age groups, should we observe a more pronounced mortality advantage among those with shorter durations than those with longer ones, then this will provide solid evidence of a duration effect.

**Fig. 3:**
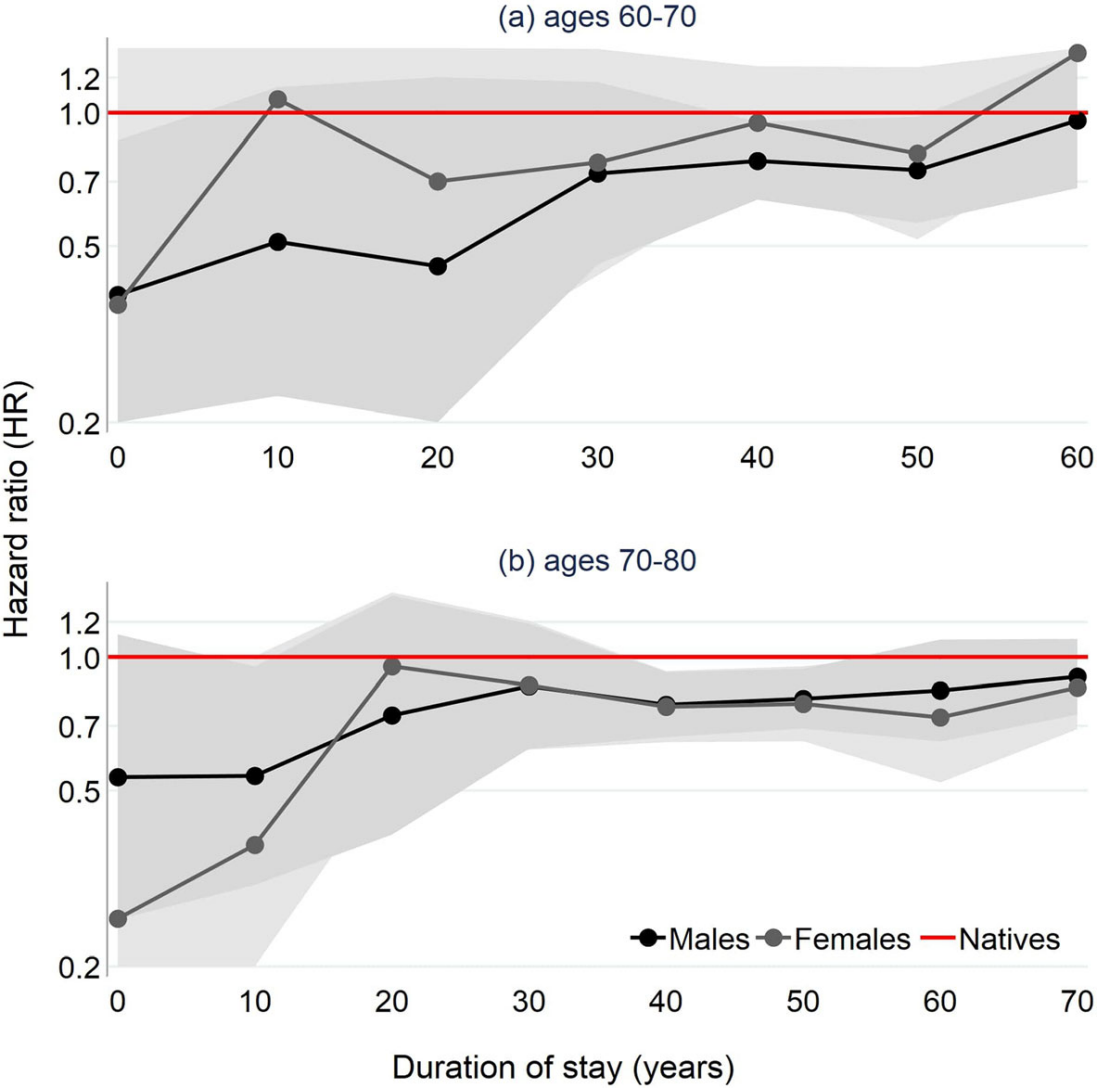
Hazard ratios (log) for all-cause mortality among male and female migrants over duration of stay, fixed at age bands 60–70 (top) and 70–80 (bottom). *Survival analyses adjusted for age, baseline year of entry, education level, and marital status. See Tables S3 and S4 of Additional file* *1* *for regression tables*

Figure 3 presents HRs for the two age groups (baseline and final models are in the Additional file 1: Tables S3 and S4). For both sexes, we continue to observe convergence in HRs with increasing duration of stay, even after fixing age. The continued presence of convergence at these older ages is promising and in both age groups is well established among males (but less so among females). For example, track the HR of males aged 60–70 who have arrived in the past 10-years (**HR = 0.39**; *95% CIs 0.17–0.87*) to that of males who arrived 60 years ago (**HR = 0.96**; *95% CIs 0.68–1.36*). Similarly, track the HR of males aged 70–80 who have arrived in the past 10-years (**HR = 0.54**; *95% CIs 0.26–1.13*) to that of males who arrived 70 years ago (**HR = 0.91**; *95% CIs 0.74–1.09*). For females, the greater fluctuation we observe (in fact in both Fig. 1 and Fig. 2) is likely explained by a lower number of death events, particularly at shorter durations (note the wider CIs relative to males and number of deaths in Tables S5 to S7 in the Additional file 1). Nonetheless, for both sexes, the very low HRs for the most recent arrivals (and therefore the size of mortality differences between migrants and natives) remain quite striking, especially considering age is fixed in categories where variability in mortality tends to be lower. Post-estimation tests show that in model **a** (ages 60–70) mortality in the shortest duration band is statistically significantly different from the longest duration band for males (**HR = 2.48**; *p* < 0.05) and marginally significant for females (**HR = 3.71**; *p* < 0.10). In model **b** (ages 70–80), the test is insignificant for males (**HR = 1.69**; *p* > 0.10) but marginally significant for females (**HR = 3.33**; *p* < 0.10).

The additional compositional characteristics in Additional file 1: Table S7 (we do not show age, age at arrival or year of arrival given age is fixed) show that migrants in the most recent duration categories originate largely from countries in the European Union (particularly Great Britain, Germany, Holland, and Belgium). Most notably, over 60% of the duration of stay category 0–10 is composed of migrants arriving from Great Britain. Additionally, the individuals in the two most recent duration of stay categories are remarkably highly qualified relative to natives and individuals in the other duration of stay categories (again, education level is adjusted for in the models). We elaborate upon these interesting findings in the discussion. One thing that we must bear in mind for the two longest duration categories is the effect of age at arrival and its association with migration selection effects (or lack thereof among migrants arriving as children who almost exclusively comprise these groups). In short, are the converged values we observe a consequence of the long time migrants have spent living in France, or because they arrived as children and were not positively selected in the first place? This remains an open-ended question for future investigation.

## Discussion

To the best of our knowledge, for the first time in France we have observed a *migrant mortality advantage* that was strongest among the most recent arrivals and then converged towards the mortality of the native population with duration of stay. Importantly, we showed this pattern to be robust to the confounding effect of age, and to some extent age at arrival, and found it to be consistent among male and female migrants. Such patterns adhere to the theorized narrative of erosion over time of the health advantage generated by initial selection, which does not provide lifelong protection. The patterns also accord with the idea of exposure to adverse conditions and a gradual and negative acculturation to the prevailing health behaviors of the host society, although we cannot favor either process with the data we have available. Our findings complement those from a French longitudinal study examining changes in diet and physical activity among Tunisian migrants [41] and the wider international literature examining acculturation in migrant health behaviors [42–48].

Contrary to our expectations, the pattern of advantage followed by convergence presented in both male and female migrants and there was no difference between the two. This challenges the perception in the literature that the *migrant mortality advantage* is gendered. In high-income countries, female migrants can be admitted as dependent wives or as independent woman integrated into the workforce [34]. This dependent/independent balance in all likelihood varies depending upon the composition of the migrant population. Notably, we should consider origin country (especially its level of gender equality and partnership norms) and year of arrival (given improving gender equality over time in both the origin and host countries leading to improved access to education and greater diversity in labor market opportunities). Additional file 1: Tables S5 and S6 provides the composition of migrant females and males by country of birth, sex, year at arrival and age at arrival. Women arriving in the past 10 years are highly qualified (over 50% have tertiary level education) and their education level distributions across duration bands are comparable with males (albeit with somewhat higher levels having primary or less in the longer duration bands). Women arriving recently do so from countries associated with skilled labor or education migration to France (Other Europe [largely from the EU], Algeria, and Sub-Saharan Africa). Furthermore, the initial mortality advantage observed among women was quite similar to that experienced by males and, unlike males, did not fully converge with rising duration of stay. This should be considered all the more striking in light of the double discrimination faced by female migrants in many host countries (as both women and ethnic minorities) [34] which presents an additional factor in the negative assimilation process which is not experienced by males.

It is fascinating that we continue to observe marked mortality advantages among migrants arriving between ages 60 and 80, especially given that mortality differences between natives and migrants should be smaller at these ages as the variability in mortality is lower. Additional file 1: Table S7 showed that migrants aged 60 to 80 who had arrived recently to live in France were highly educated and originated largely from other European nations, particularly Great Britain. With this in mind, we offer two tentative explanations for this old age migrant mortality advantage. For migrants arriving at pre-retirement age (retirement age in France depends upon date of birth: some can retire from 60; everyone must retire by 70 [49]) most will still be moving for work and will continue to be subjected to the selection processes associated with the *healthy migrant* and *healthy worker effects*. France’s reunification policy relates only to spouses and children; not the elderly parents of migrants [50]. For migrants arriving post-retirement, our explanation relates to international retirement migration (IRM) [51]. France is a popular host country for IRM, especially for British citizens [who comprise a staggering 60% of the duration category 0–10 for males and females]. IRM is socially selective, most of those who move countries to retire are either ‘early retired’ or ‘active young’ elderly with unique levels of wealth and income (and so presumably good health) [51]. The mortality risk of international retirement migrants is not likely to be reflective of individuals of the same age they move to live amongst.

## Conclusions

The main strength of this study lies in the use of a large and representative longitudinal data source to investigate detailed variation in migrant mortality over duration of stay, with a particular emphasis on the roles of age and sex. The main limitation of the study is that we were unable to make full use of the entire EDP data. Consequently, we were unable to investigate specific variation by the country of birth or education level of migrants, which could have provided fresh insight into the two primary explanations of the migrant mortality advantage. Nonetheless, the main contribution of this study has been to isolate an intrinsic duration of stay effect in patterns of migrant mortality. Our findings should promote renewed interest in the experiences of migrants after they arrive in the host country, as it is vital to determine whether the convergence we have observed is an unavoidable one (selection effects wearing off) or accelerated by the lifelong hardship to which migrants can be exposed in the host country. In the latter case, targeted health policies would be needed to preserve the initial substantial health capital of migrants and prevent (or slow) this erosion. Achieving this goal would help to maximize the potential social, cultural, and economic contributions of migrants and support their healthy ageing. Future studies could investigate how the effect of duration varies over country of birth and other socio-demographic characteristics and give more salience to gender dynamics and equality in countries of origin. Additionally, examining how causes of death vary over duration of stay in relation to all-cause mortality would provide crucial insight into acculturation and the migrant mortality advantage.

## Additional file

Additional file1: **Table S1.** Hazard ratios (log) for all-cause mortality among male migrants over duration of stay, ages 20+, baseline and final models. (1) baseline year of entry adjusted for but not shown (2) significant levels at ** p < 0.01, * p < 0.05, and + p < 0.10. **Table S2.** Hazard ratios (log) for all-cause mortality among female migrants over duration of stay, ages 20+, baseline and final models. (1) baseline year of entry adjusted for but not shown (2) significant levels at ** p < 0.01, * p < 0.05, and + p < 0.10. **Table S3.** Hazard ratios (log) for all-cause mortality among male and female migrants over duration of stay, fixed at age bands 60–70, baseline and final models. (1) baseline year of entry adjusted for but not shown (2) significant levels at ** p < 0.01, * p < 0.05, and + p < 0.10. **Table S4.** Hazard ratios (log) for all-cause mortality among male and female migrants over duration of stay, fixed at age bands 70–80, baseline and final models. (1) baseline year of entry adjusted for but not shown (2) significant levels at ** p < 0.01, * p < 0.05, and + p < 0.10. **Table S5.** Compositional characteristics (education, country of origin, year and age of arrival, and age) of male migrants over duration of stay, ages 20+. **Table S6.** Compositional characteristics (education, country of origin, year and age of arrival, and age) of female migrants over duration of stay, ages 20+. **Table S7.** Compositional characteristics (education and country of origin only) of male and female migrants over duration of stay, ages 60–80. (XLSX 43 kb)

## Abbreviations

*CIs*: Confidence Intervals
*EDP*: *Échantillon Démographique Permanent*
*HRs*: Hazard Ratios
*IRM*: International Retirement Migration
*ISCED*: International Standard Classification of Education
*OR*: Odds Ratios
*UK*: United Kingdom
*US*: United States
